# Association of CD44 gene rs187115 polymorphism with colorectal cancer risk and prognosis in Chinese Han population: a case-control study

**DOI:** 10.18632/aging.102408

**Published:** 2019-11-03

**Authors:** Qian Wan, Dan Zhang, Qing Zhou, Ming Li, Yujuan Wang, Yang Song, Tianshu Xu

**Affiliations:** 1 Department of Traditional Chinese Medicine, Nanjing Drum Tower Hospital, The Affiliated Hospital of Nanjing University Medical School, Nanjing, China; 2 Department of Anorectal Surgery, Jiangsu Provincial Hospital of Traditional Chinese Medicine, Nanjing, Jiangsu, China

**Keywords:** CD44, colorectal cancer, case-control study, rs187115 polymorphism, prognosis

## Abstract

The cell surface adhesion receptor CD44 reportedly affects the development and progression of cancers. Moreover, CD44 gene rs187115 polymorphism appears to be genetic determinant of cancer susceptibility. We investigated whether CD44 rs187115 polymorphism is associated with colorectal cancer (CRC) risk and prognosis. We enrolled 669 CRC cases and 826 controls in this three-center case-control study in a Chinese Han population. All individuals were genotyped by matrix-assisted laser desorption/ionization time-of-flight mass spectrometry. Cross-over analysis, multivariate logistic regression, Kaplan-Meier method, and Cox regression analysis were used for analysis. In this study, CD44 rs187115 polymorphism was associated with increased risk for CRC. Stratified analyses revealed that CD44 rs187115 polymorphism was correlated with increased risk for CRC in females, drinkers, smokers, and those aged ≥ 60 years. In addition, rs187115 polymorphism was significantly associated with TNM III+IV stage, lymph node metastasis and tumor size in CRC patients. Combined effects of CD44 rs187115 polymorphism (GG/AG vs. AA) and environmental factors (smoking and drinking) further increased the risk of CRC. GG genotype carriers showed poorer overall survival than AA genotype carriers. Cox regression analysis showed that drinking, CD44 rs187115 polymorphism, and TNM stage were independent prognostic factors affecting CRC. These findings show that CD44 rs187115 polymorphism may be a potential biomarker predictive of CRC susceptibility and prognosis.

## INTRODUCTION

Colorectal cancer (CRC) is among the most commonly diagnosed cancers and a major cause of death from cancer worldwide [1]. For example, more than 1.8 million CRC cases and 881,000 related deaths were reported in 2018 [1]. In the United States, 135, 000 new CRC cases and 49, 000 CRC-related deaths were estimated in 2017 [2]. In China, CRC is the 4^th^ most common cancer among women and the 5^th^ most common among men [3]. The most important factor affecting the prognosis of CRC patients is early detection; while tumors are still localized, the 5-year survival rate is > 95% [4].

The pathogenesis of CRC is still unclear. Cigarette smoking [5], drinking [6, 7], dietary patterns [8, 9] are all risk factors for CRC. It is well known that interactions between genetic factors and environmental factors contribute to the occurrence and development of CRC [10]. Identifying new genetic biomarkers for diagnosis and prediction related to CRC would be of great clinical relevance.

The cell-surface glycoprotein CD44 is a major adhesion molecule within the extracellular matrix. By functioning as a receptor for osteopontin and hyaluronate, CD44 mediates cellular adhesion to cell-extracellular matrix [11]. CD44 also plays important roles in the differentiation, invasion and metastasis of tumor cells [12, 13], is associated with poor clinical outcomes in cancer patients [14, 15]. The CD44 gene is located on chromosome 11p13 [16]. Single-nucleotide polymorphisms (SNPs) in CD44 gene may affect the protein expression, thereby influencing individuals’ susceptibility to cancer. In particular, a number of studies [17–25] have explored the association between the CD44 rs187115 polymorphism and cancer risk, though their findings have been inconsistent. One Chinese study [22] found that CD44 rs187115 polymorphism was not associated with CRC risk in a southern Chinese population. To assess this relationship in an eastern Chinese population, we designed a three-center case-control study to evaluate the association between CD44 rs187115 polymorphism and CRC risk. In addition, we explored the link between CD44 rs187115 polymorphism and the clinical features of CRC and its prognosis.

## RESULTS

### Characteristics of the study population

The baseline characteristics of the participants are summarized in Table 1. There were no significant differences between the cases and controls with regard to age, sex and smoking habits. Notably, the distributions of drinkers in the cases differed from those in the controls (*P* <0.001). Among the 669 patients included in the study, 461 had colon cancer and 208 had rectal cancer. Among the CRC cases, 643 (96.1%) were adenocarcinomas, 18 (2.7%) were squamous cell carcinomas, and 8 (1.2%) were other types of CRC. Sixty-nine (10.3%) of the tumors were well differentiated, 517 (77.3%) were moderately differentiated, and 83 (12.4%) were poorly differentiated. Other clinical parameters investigated were TNM stage, lymph node metastasis, family history, and tumor size.

**Table 1:** Patient demographics and risk factors in colorectal cancer.

**Characteristics**	**Case (N=669)**	**Control (N=826)**	***P***
Age	61.11±10.93	60.47±15.07	0.343
Sex			0.627
Male	347(51.9%)	418(50.6%)	
Female	322(48.1%)	408(49.4%)	
Smoking			0.215
YES	369(55.2%)	429(51.9%)	
NO	300(44.8%)	397(48.1%)	
Alcohol			<0.001
YES	463(69.2%)	474(57.4%)	
NO	206(30.8%)	352(42.6%)	
Family history			
YES	98(14.6%)		
NO	571(85.4%)		
Histological grade			
well differentiated	69(10.3%)		
Moderately differentiated	517(77.3%)		
Poorly differentiated	83(12.4%)		
TNM stage			
I+II	335(50.1%)		
III+IV	334(49.9%)		
Tumor size			
>5 cm	415(62.3%)		
≤5 cm	254(37.7%)		
Lymph node metastasis			
NO	398(59.5%)		
YES	271(40.5%)		
Histology			
Adenocarcinoma	643(96.1%)		
Squamous cell carcinoma	18(2.7%)		
Others	8(1.2%)		
Location of colorectal cancer			
colon cancer	461(68.9%)		
–rectal cancer	208(31.1%)		

### Relationship between CD44 gene rs187115 polymorphism and CRC risk

The genotype and allele distributions for CD44 gene rs187115 polymorphism differed significantly between the CRC patients and the controls (Table 2). There was no significant deviation in the genotypic frequencies from the Hardy-Weinberg equilibrium (HWE) among the controls. Individuals with AG or GG genotype were at higher risk than those carrying the AA genotype (AG vs. AA: OR, 1.74; 95%CI, 1.41-2.16; *P* = 0.002; GG vs. AA: OR, 1.88; 95%CI, 1.27-2.78; *P* = 0.002). Similarly, subjects with the GG+AG genotype or G allele were found to have a significantly increased risk for CRC. These findings also held true in the homozygote and dominant genetic models after adjusting for sex and age.

**Table 2:** Genotype frequencies of CD44 gene polymorphisms in cases and controls.

**Models**	**Genotype**	**Case (n, %)**	**Control (n, %)**	**OR (95% CI)**	***P*-value**	*OR (95% CI)	*P-value
rs187115							
Co-dominant	AA	273(40.9%)	454(55.0%)	1.00(reference)	-	1.00(reference)	
Heterozygote	AG	332(49.8%)	317(38.4%)	**1.74(1.41-2.16)**	**<0.001**	**1.63(1.31-2.02)**	<0.001
Homozygote	GG	62(9.3%)	55(6.7%)	**1.88(1.27-2.78)**	**0.002**	**1.85(1.24-2.74)**	0.002
Dominant	AA	273(40.9%)	454(55.0%)	1.00(reference)	-	1.00(reference)	
	GG+AG	394(59.1%)	372(45.0%)	**1.76(1.43-2.17)**	**<0.001**	**1.66(1.34-2.04)**	<0.001
Recessive	AG+AA	605(90.7%)	771(93.3%)	1.00(reference)	-	1.00(reference)	
	GG	62(9.3%)	55(6.7%)	1.44(0.98-2.10)	0.061	**1.47(1.00-2.15)**	0.050
Allele	A	878(65.8%)	1225(74.2%)	1.00(reference)	-	1.00(reference)	
	G	456(34.2%)	427(25.8%)	**1.49(1.27-1.75)**	**<0.001**	**-**	**-**

The genotyping was successful in 667 cases and 826 controls for rs187115;

Bold values are statistically significant (P <0.05).

*Adjust age and sex.

We then further evaluated the effect of CD44 rs187115 polymorphism on the risk of CRC after stratification based on sex, age, alcohol and smoking (Table 3). The significant relationship between CD44 rs187115 polymorphism and CRC risk was more pronounced among female, smokers, drinkers, and patients aged ≥ 60 years.

**Table 3:** Stratified analyses between rs187115 polymorphisms and the risk of colorectal cancer.

**Variable**	**(case/control)**			**AG vs. AA**	**GG vs. AA**	**GG vs. AA+AG**	**GG+AG vs. AA**
	**AA**	**AG**	**GG**				
Sex							
Male	177/230	137/158	31/30	1.13(0.83-1.53); 0.438	1.34(0.78-2.30); 0.284	1.28(0.76-2.16); 0.360	1.16(0.87-1.54); 0.306
Female	96/224	195/159	31/25	**2.86(2.08-3.93); <0.001**	**2.89(1.62-5.16); <0.001**	1.63(0.94-2.82); 0.080	**2.87(2.11-3.90); <0.001**
Smoking							
Yes	129/238	202/167	36/24	**2.23(1.66-3.00); <0.001**	**2.77(1.58-4.84); <0.001**	**1.84(1.07-3.14); 0.027**	**2.30(1.73-3.06); <0.001**
No	144/216	130/150	26/31	1.30(0.95-1.78); 0.103	1.26(0.72-2.21); 0.424	1.12(0.65-1.93); 0.682	1.29(0.96-1.75); 0.094
Alcohol							
Yes	159/266	253/180	49/28	**2.35(1.79-3.09); <0.001**	**2.93(1.77-4.85); <0.001**	**1.89(1.17-3.07); 0.010**	**2.43(1.87-3.16); <0.001**
No	114/188	79/137	13/27	0.95(0.66-1.37); 0.785	0.79(0.39-1.60); 0.519	0.81(0.41-1.61); 0.549	0.93(0.66-1.31); 0.659
Age (years)							
<60	162/290	121/184	32/35	1.18(0.87-1.59); 0.285	1.64(0.98-2.74); 0.062	1.53(0.93-2.53); 0.096	1.25(0.94-1.66); 0.120
≥60	111/164	211/133	30/20	**2.34(1.70-3.24); <0.001**	**2.22(1.20-4.10); 0.011**	1.38(0.77-2.49); 0.279	**2.33(1.70-3.19); <0.001**

Bold values are statistically significant (*P* <0.05).

### Combined and interactive effects of polymorphisms and environmental factors on the risk of CRC

We used cross-over analysis to evaluate the effects of the interaction between genetic factors and smoking or drinking on CRC risk (Table 4). For non-smokers and non-drinkers, the AG or GG genotype is not associated with a greater risk of CRC as compared to the AA genotype. For smokers, however, carrying the GG or AG genotype was significantly associated with an increased risk of CRC as compared to non-smokers carrying AA genotypes (GG + smoking vs. AA + non-smoking: OR, 2.25, 95%CI, 1.29-3.93; *P* = 0.004; AG + smoking vs. AA + non-smoking: OR, 1.81, 95%CI, 1.35-2.44; *P* < 0.001). Similarly, CRC patients who drank alcohol and carried the GG or AG genotype had a higher risk of developing CRC than patients who did not drink alcohol and carried the AA genotype (GG + drinking vs. AA + non- drinking: OR, 2.89, 95%CI, 1.72-4.85; *P* < 0.001; AG + drinking vs. AA + non- drinking: OR, 2.32, 95%CI, 1.72-3.13; *P* < 0.001). We found that significant combined effects of CD44 rs187115 polymorphism and drinking contributed to an increased risk of CRC (Table 5). This indicates that there is a potential interaction between genetic factors and environment factors in CRC.

**Table 4:** Genetic (G) and environmental (E) factors 2*4 fork analysis.

**G^a^**	**E^b^**	**Case**	**Control**	**OR (95%CI); P value**	**Reflecting information**
GG/AA	Smoking				
+	+	36	24	**2.25(1.29,3.93); 0.004**	G (+), the role of E
+	-	26	31	1.26(0.72,2.21); 0.423	G (-), the role of E
-	+	129	238	0.81(0.60,1.10); 0.177	E (+), the role of G
-	-	144	216	1.00	E (-), the role of G
AG/AA	Smoking				
+	+	202	167	**1.81(1.35,2.44); <0.001**	G (+), the role of E
+	-	130	150	1.30(0.95,1.78); 0.103	G (-), the role of E
-	+	129	238	0.81(0.60,1.10); 0.177	E (+), the role of G
-	-	144	216	1.00	E (-), the role of G
GG/AA	Drinking				
+	+	49	28	**2.89(1.72,4.85); <0.001**	G (+), the role of E
+	-	13	27	0.79(0.39,1.60); 0.519	G (-), the role of E
-	+	159	266	0.99(0.73,1.34); 0.926	E (+), the role of G
-	-	114	188	1.00	E (-), the role of G
AG/AA	Drinking				
+	+	253	180	**2.32(1.72,3.13); <0.001**	G (+), the role of E
+	-	79	137	0.95(0.66,1.37); 0.785	G (-), the role of E
-	+	159	266	0.99(0.73,1.34); 0.926	E (+), the role of G
-	-	114	188	1.00	E (-), the role of G

^a^G (+): GG orAG genotypes of CD44 gene rs187115 polymorphism; G (-): AA genotype.

^b^E(+): smoking/drinking; E(-): non-smoking/non-drinking.

**Table 5:** The associations between CD44 rs187115 polymorphism and clinical characteristics of colorectal cancer.

**Characteristics rs2275913**	**Genotype distributions**			
	**AA**	**AG**	**GG**	**AG+GG**
Histological grade				
MD/WD	218/27	251/34	46/8	297/42
OR (95%CI); *P*-value	1.0 (reference)	0.91(0.54-1.56); 0.744	0.71(0.30-1.67); 0.434	0.88(0.52-1.46); 0.613
Histological grade				
PD/WD	28/27	47/34	8/8	55/42
OR (95%CI); *P*-value	1.0 (reference)	1.33(0.67-2.65); 0.413	0.96(0.32-2.94); 0.949	1.26(0.65-2.45); 0.491
TNM stage				
III+IV/I+II	120/153	175/157	38/24	213/181
OR (95%CI); *P*-value	1.0 (reference)	**1.42(1.03-1.96); 0.032**	**2.02(1.15-3.55); 0.015**	**1.50(1.10-2.05); 0.010**
Tumor size				
>5 cm/≤5 cm	154/119	216/116	45/17	261/133
OR (95%CI); *P*-value	1.0 (reference)	**1.44(1.04-2.00); 0.030**	**2.05(1.12-3.75); 0.021**	**1.52(1.10-2.08); 0.010**
Lymph node metastasis				
YES/NO	95/178	144/188	30/32	174/220
OR (95%CI); *P*-value	1.0 (reference)	**1.44(1.03-2.00); 0.032**	**1.76(1.01-3.07); 0.047**	**1.48(1.08-2.04); 0.016**
Family history				
YES/NO	36/237	52/280	10/52	62/332
OR (95%CI); *P*-value	1.0 (reference)	1.22(0.77-1.93); 0.391	1.27(0.59-2.71); 0.544	1.23(0.79-1.92); 0.361
Histology				
Adenocarcinoma/NOT	260/13	322/10	59/3	381/13
OR (95%CI); *P*-value	1.0 (reference)	1.61(0.70-3.73); 0.267	0.98(0.27-3.56); 0.980	1.47(0.67-3.21); 0.340
Location of colorectal cancer				
colon cancer/ rectal cancer	182/91	231/101	46/16	277/117
OR (95%CI); *P*-value	1.0 (reference)	1.14(0.81-1.61); 0.444	1.44(0.77-2.68); 0.253	1.18(0.85-1.65); 0.319

Bold values are statistically significant (*P* <0.05). PD = Poorly differentiation, MD= Moderately differentiation, WD= Well differentiation.

### Correlation between CD44 rs187115 polymorphism and the clinicopathological characteristics of CRC patients

We next assessed the relationship between CD44 rs187115 polymorphism and the clinicopathological characteristics of CRC patients. As shown in Table 5, the rs187115 AG or GG genotype was more frequent in patients with higher TNM stage (III+IV), larger tumor size (> 5 cm) and lymph node metastasis. In other words, CD44 rs187115 polymorphism was associated with TNM stage, tumor size and lymph node metastasis in CRC.

### CD44 rs187115 polymorphism with CRC patient prognosis

**Figure 1:**
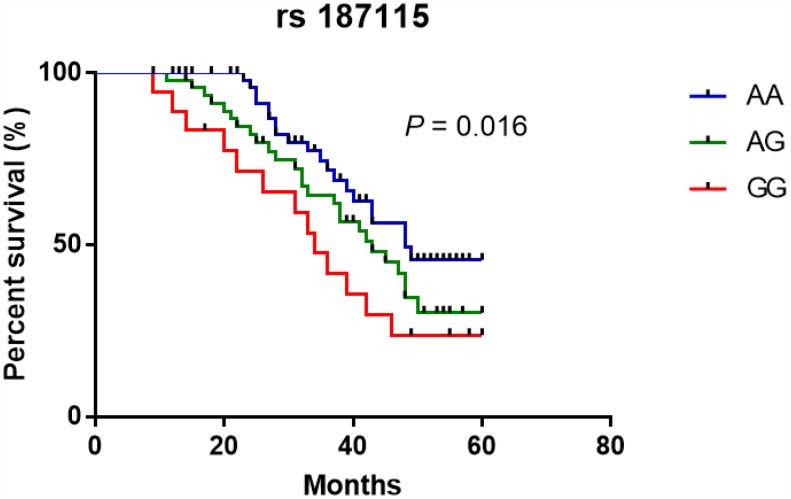
**Kaplan-Meier analysis of the effect of CD44 rs187115 polymorphism on overall survival among CRC patients.**

We followed up 125 CRC patients to evaluate the effect of CD44 rs187115 polymorphism on the risk of CRC development (Table 6). Patients with the GG or AG genotype showed lower overall survival than those carrying the AA genotype (Figure 1). Univariate analysis indicated that rs187115 genotype (GG vs. AA, HR, 2.11, 95%CI, 1.03-4.31; *P* = 0.042), drinking (HR, 1.71, 95%CI, 1.01–2.90; *P* = 0.045), TNM stage (HR, 1.97, 95%CI, 1.17–3.30; *P* = 0.011), and tumor size (HR, 1.86, 95%CI, 1.11–3.12; *P* = 0.019) were associated with prognosis in CRC (Table 6). Subsequent multivariate analysis confirmed that drinking, CD44 rs187115 polymorphism, and TNM stage were independent prognostic factors in CRC patients.

**Table 6:** Prognostic factors in Cox proportional hazards model for colorectal cancer.

**Variables**	**Univariate analysis**			**Multivariate analysis**		
	**Hazard ratio**	**95% CI**	***P* value**	**Hazard ratio**	**95% CI**	***P* value**
Age, years						
≥60/<60	1.32	0.77-2.26	0.321	1.39	0.78-2.49	0.268
Gender						
Male/female	0.74	0.44-1.26	0.743	0.93	0.50-1.73	0.825
Smoking						
Yes/No	1.14	0.68-1.90	0.621	1.28	0.72-2.27	0.406
Drinking						
Yes/No	**1.71**	**1.01-2.90**	**0.045**	**2.02**	**1.10-3.69**	**0.023**
Histological grade						
MD/WD	0.48	0.22-1.08	0.075	0.62	0.24-1.60	0.325
Histological grade						
PD/WD	1.12	0.42-3.01	0.825	1.04	0.33-3.27	0.951
TNM stage						
III+IV/I+II	**1.97**	**1.17-3.30**	**0.011**	**2.06**	**1.13-3.74**	**0.019**
Tumor size						
>5 cm/≤5 cm	**1.86**	**1.11-3.12**	**0.019**	1.48	0.83-2.67	0.188
Lymph node metastasis						
YES/NO	0.96	0.51-1.81	0.896	0.72	0.34-1.52	0.382
Histology						
Adenocarcinoma/NOT	1.38	0.34-5.68	0.651	0.99	0.22-4.42	0.991
Location of colorectal cancer						
Colon cancer/ rectal cancer	1.17	0.68-2.02	0.578	1.34	0.70-2.57	00.375
Genotype						
AG/AA	1.60	0.90-2.85	0.111	**2.15**	**1.14-4.07**	**0.019**
GG/AA	**2.11**	**1.03-4.31**	**0.042**	**3.11**	**1.37-7.09**	**0.007**

Bold values are statistically significant (*P* <0.05). PD = Poorly differentiation, MD= Moderately differentiation, WD= Well differentiation.

## DISCUSSION

In this study, we found that CD44 rs187115 polymorphism was associated with increased risk of CRC in this eastern Chinese population. Stratified analyses showed CD44 rs187115 polymorphism to be related to increased risk for CRC in male, drinkers, smokers, and older (≥ 60 years) individuals. Furthermore, this SNP was significantly correlated with TNM III+IV stage, lymph node metastasis and tumor size (>5 cm) in CRC patients. Cox regression analysis revealed that drinking, CD44 rs187115 polymorphism, and TNM stage were independent prognostic factors for CRC patients.

To date, several studies have investigated the association between CD44 rs187115 polymorphism and cancer risk with conflicting findings. Vazquez et al. found that CD44 rs187115 polymorphism was associated with weaker responses to chemotherapeutics and with poorer overall survival with soft-tissue sarcomas [17]. A subsequent study from Taiwan revealed that the gene-environment interactions between CD44 rs187115 polymorphism and betel quid chewing and smoking increase the risk of oral cancer [18]. Two other Taiwanese studies showed that this SNP was related to an increased risk and poor prognosis in bladder cancer [19] and hepatocellular carcinoma [20]. However, a study from China showed that CD44 rs187115 polymorphism was associated with a decreased risk for non-small cell lung cancer (NSCLC), but was also significantly related with bone metastasis and tumor stage for NSCLC patients [21]. Those investigators suggested this SNP might become a potential prognostic marker in NSCLC patients [21]. In another Chinese study [22], Chen et al. observed that rs187115 polymorphism was associated with the risk of cervical, lung, and liver cancer, but not with the risk of breast, gastric, colon or rectal cancer. In Indian populations, however, no significant association was found with gallbladder [23, 24] and bladder cancer patients [25]. In this present study, we found CD44 rs187115 polymorphism was associated with increased risk for CRC, which is different from the study by Chen et al [22]. We suggest the reasons for that discrepancy are as follows. First, exposure factors were different between these two studies. Second, the eating habits and living environments were distinct. Third, the clinical heterogeneity of CRC may also be a potential factor. And fourth, the sample sizes differed.

In the stratified analyses, data indicated CD44 rs187115 polymorphism was associated with increased CRC risk in drinkers, smokers, and older (≥ 60 years) individuals, indicating potential interactions between these expose risk factors and CD44 rs187115 polymorphism, which may contribute to an increased risk of CRC. Thus, we next used the cross-over analysis to evaluate the effects of these abovementioned factors on CRC susceptibility. Data suggested that the combined effects of CD44 gene rs187115 polymorphism and drinking were partly account for an increased risk of CRC (Table 4). However, no significant interaction between rs187115 polymorphism and smoking was observed. Next, this study addressed the association between rs187115 polymorphism and clinical characteristics of CRC patients. We found that the rs187115 polymorphism genotype carriers in CRC patients were associated with lymph node metastasis, tumor size > 5 cm, and TNM III+IV stage. In previous studies, Stotz et al. showed that rs187115 polymorphism in the stem cell gene CD44 predicted the outcomes in Stage II and Stage III colon cancer patients [26]. They indicated that CD44 rs187115 polymorphism showed a statistically significant association with recurrence [26], suggesting a risky role of this SNP in CRC patients, which was consistent with this study. However, Ramasami et al. indicated that CD44 was not associated with lymph node metastases or tumor stage [27].

We further evaluated the relationship between CD44 rs187115 polymorphism and CRC patient prognosis. 125 CRC patients were followed up to assess the effects of CD44 rs187115 polymorphism on the risk of CRC. Data indicated that AG+GG genotype carriers yielded poorer overall survival compared with AA genotype carriers. Cox regression analysis showed that drinking, CD44 rs187115 polymorphism, and TNM stage were independent prognostic factors in CRC patients, which was consistent with the findings by Liu et al. [21] that rs187115 polymorphism a poor prognostic factor for lung cancer. Vazquez et al. revealed that CD44 rs187115 polymorphism was related to increased risk for sarcoma-related death and lower drug sensitivity [17]. All these data suggested that CD44 rs187115 polymorphism may be a prognostic factor for different cancers.

There are several potential limitations to the present study. First, the sample size was relatively small. Second, selection bias was unavoidable in this case-control study. Third, the follow-up data on CRC patients were deficient in some cases. Fourth, the two populations may not represent the general population because only hospital patients were enrolled. Fifth, we only explored one SNP in the CD44 gene. Finally, we could not provide data from experiments with cells or mice that could shed light on the mechanisms responsible for why the rs187115 polymorphism is associated with an increased CRC risk and a poorer prognosis for CRC patients. Consequently, those mechanisms remain unclear.

In conclusion, CD44 rs187115 polymorphism is associated with increased risk and prognosis in CRC. Further studies with a larger, more diverse population will be needed to determine whether the involvement of the CD44 rs187115 polymorphism can be generalized to a broader population.

## MATERIALS AND METHODS

### Subjects

In this three-center case–control study, 669 CRC patients and 826 sex- and age-matched controls were recruited from the Jiangsu Provincial of Traditional Chinese Medicine, Nanjing Drum Tower Hospital, and the Third Affiliated Hospital of Nantong University. All CRCs were confirmed histopathologically. The clinicopathologic characteristics of the CRC patients were extracted from medical records. The data collected included age, sex, smoking, alcohol intake, histological grade, family history, TNM stage, lymph node metastasis, tumor size, location of CRC, and the histology. Eligible controls were recruited from the same region as the cases during the same period.

This study was approved by the ethics committees of the Jiangsu Provincial of Traditional Chinese Medicine, Nanjing Drum Tower Hospital and the Third Affiliated Hospital of Nantong University and met the standards of the Declaration of Helsinki. Written informed consent was obtained from all the participants, and all the participants were above the age of 16.

### Blood sampling and genotyping

DNA was extracted from peripheral blood leukocytes collected from the study participants using a DNA Purification Kit (Tiangen Biotech) according to the manufacturer’s instructions [28]. Genotyping was done with matrix-assisted laser desorption/ionization time-of-flight mass spectrometry (MALDI-TOFMS) using a MassARRAY system (Sequenom, San Diego, CA, USA). The primers used for nucleotide extension reaction were AGAAAGATGTTATCATCGTCACTG (forward) and GATGCCTTCAGATGCGAGTA (reverse). About 10% of the selected samples were randomly chosen for genotyping twice to ensure the genotyping accuracy. The results were 100% concordant.

### Statistical analysis

All statistical analyses were conducted using SPSS 22.0 (SPSS Inc., Chicago, USA). The variables for cases and controls were estimated using Student’s t-tests (continuous variables) or Chi-square (χ^2^) tests (categorical variables). HWE was evaluated using a χ^2^-test. Using logistic regression analysis, the allele type and genotype distributions in two groups were evaluated by computing the crude and adjusted odds ratios (ORs) and their 95% confidence intervals (CIs). Stratified analyses were conducted according to sex, age, alcohol and smoking status. Logistic regression analyses were also used to evaluate the exposure combination models. Overall survival was calculated from the date of surgery to the time of death using the Kaplan-Meier method. We also examined the potential effects of different intervening variables (sex, age, smoking, drinking, histological grade, TNM stage, tumor size, lymph node metastasis, histology, genotype of rs187115 polymorphism and location of CRC) on survival of CRC patients using a multivariate Cox proportional hazards method (Backward Wald). Values of *P* < 0.05 were considered statistically significant.

## References

[1] Bray F, Ferlay J, Soerjomataram I, Siegel RL, Torre LA, Jemal A. Global cancer statistics 2018: GLOBOCAN estimates of incidence and mortality worldwide for 36 cancers in 185 countries. CA Cancer J Clin. 2018; 68:394–424. 10.3322/caac.21492 30207593

[2] Lieberman D, Ladabaum U, Cruz-Correa M, Ginsburg C, Inadomi JM, Kim LS, Giardiello FM, Wender RC. Screening for Colorectal Cancer and Evolving Issues for Physicians and Patients: A Review. JAMA. 2016; 316:2135–45. 10.1001/jama.2016.17418 27893135

[3] Chen W, Zheng R, Baade PD, Zhang S, Zeng H, Bray F, Jemal A, Yu XQ, He J. Cancer statistics in China, 2015. CA Cancer J Clin. 2016; 66:115–32. 10.3322/caac.21338 26808342

[4] Etzioni R, Urban N, Ramsey S, McIntosh M, Schwartz S, Reid B, Radich J, Anderson G, Hartwell L. The case for early detection. Nat Rev Cancer. 2003; 3:243–52. 10.1038/nrc1041 12671663

[5] Yang C, Wang X, Huang CH, Yuan WJ, Chen ZH. Passive Smoking and Risk of Colorectal Cancer: A Meta-analysis of Observational Studies. Asia Pac J Public Health. 2016; 28:394–403. 10.1177/1010539516650724 27217428

[6] Cai S, Li Y, Ding Y, Chen K, Jin M. Alcohol drinking and the risk of colorectal cancer death: a meta-analysis. Eur J Cancer Prev. 2014; 23:532–9. 10.1097/CEJ.0000000000000076 25170915

[7] Fedirko V, Tramacere I, Bagnardi V, Rota M, Scotti L, Islami F, Negri E, Straif K, Romieu I, La Vecchia C, Boffetta P, Jenab M. Alcohol drinking and colorectal cancer risk: an overall and dose-response meta-analysis of published studies. Ann Oncol. 2011; 22:1958–72. 10.1093/annonc/mdq653 21307158

[8] Song M, Chan AT, Fuchs CS, Ogino S, Hu FB, Mozaffarian D, Ma J, Willett WC, Giovannucci EL, Wu K. Dietary intake of fish, omega-3 and omega-6 fatty acids and risk of colorectal cancer: A prospective study in U.S. men and women. Int J Cancer. 2014; 135:2413–23. 10.1002/ijc.28878 24706410 PMC4159425

[9] Castelló A, Amiano P, Fernández de Larrea N, Martín V, Alonso MH, Castaño-Vinyals G, Pérez-Gómez B, Olmedo-Requena R, Guevara M, Fernandez-Tardon G, Dierssen-Sotos T, Llorens-Ivorra C, Huerta JM. Low adherence to the western and high adherence to the mediterranean dietary patterns could prevent colorectal cancer. Eur J Nutr. 2018; 58:1495–1505. 10.1007/s00394-018-1674-5 29582162

[10] Balavarca Y, Weigl K, Thomsen H, Brenner H. Performance of individual and joint risk stratification by an environmental risk score and a genetic risk score in a colorectal cancer screening setting. Int J Cancer. 2019. [Epub ahead of print]. 10.1002/ijc.32272 30868574

[11] Arch R, Wirth K, Hofmann M, Ponta H, Matzku S, Herrlich P, Zöller M. Participation in normal immune responses of a metastasis-inducing splice variant of CD44. Science. 1992; 257:682–5. 10.1126/science.1496383 1496383

[12] Hill A, McFarlane S, Johnston PG, Waugh DJ. The emerging role of CD44 in regulating skeletal micrometastasis. Cancer Lett. 2006; 237:1–9. 10.1016/j.canlet.2005.05.006. 15979783

[13] Wang Z, Zhao K, Hackert T, Zoller M. CD44/CD44v6 a Reliable Companion in Cancer-Initiating Cell Maintenance and Tumor Progression. Front Cell Dev Biol. 2018; 6:97. 10.3389/fcell.2018.00097 30211160 PMC6122270

[14] Brown RL, Reinke LM, Damerow MS, Perez D, Chodosh LA, Yang J, Cheng C. CD44 splice isoform switching in human and mouse epithelium is essential for epithelial-mesenchymal transition and breast cancer progression. J Clin Invest. 2011; 121:1064–74. 10.1172/JCI44540 21393860 PMC3049398

[15] Luo Z, Wu RR, Lv L, Li P, Zhang LY, Hao QL, Li W. Prognostic value of CD44 expression in non-small cell lung cancer: a systematic review. Int J Clin Exp Pathol. 2014; 7:3632–46. 25120740 PMC4128975

[16] Goodfellow PN, Banting G, Wiles MV, Tunnacliffe A, Parkar M, Solomon E, Dalchau R, Fabre JW. The gene, MIC4, which controls expression of the antigen defined by monoclonal antibody F10.44.2, is on human chromosome 11. Eur J Immunol. 1982; 12:659–63. 10.1002/eji.1830120807 7140811

[17] Vazquez A, Grochola LF, Bond EE, Levine AJ, Taubert H, Müller TH, Würl P, Bond GL. Chemosensitivity profiles identify polymorphisms in the p53 network genes 14-3-3tau and CD44 that affect sarcoma incidence and survival. Cancer Res. 2010; 70:172–80. 10.1158/0008-5472.CAN-09-2218 19996285

[18] Chou YE, Hsieh MJ, Hsin CH, Chiang WL, Lai YC, Lee YH, Huang SC, Yang SF, Lin CW. CD44 gene polymorphisms and environmental factors on oral cancer susceptibility in Taiwan. PloS one. 2014; 9:e93692. 10.1371/journal.pone.0093692 24699672 PMC3974805

[19] Weng WC, Huang YH, Yang SF, Wang SS, Kuo WH, Hsueh CW, Huang CH, Chou YE. Effect of CD44 gene polymorphisms on risk of transitional cell carcinoma of the urinary bladder in Taiwan. Tumour Biol. 2016; 37:6971–7. 10.1007/s13277-015-4566-9 26662954

[20] Chou YE, Hsieh MJ, Chiou HL, Lee HL, Yang SF, Chen TY. CD44 gene polymorphisms on hepatocellular carcinoma susceptibility and clinicopathologic features. Biomed Res Int. 2014; 2014:231474. 10.1155/2014/231474 24971320 PMC4058263

[21] Liu Y, Qing H, Su X, Wang C, Li Z, Liu S. Association of CD44 Gene Polymorphism with Survival of NSCLC and Risk of Bone Metastasis. Med Sci Monit. 2015; 21:2694–700. 10.12659/MSM.894357 26356590 PMC4573070

[22] Chen B, Yi C, Wang J, Wang J, Zhang J, Gu X, Feng X. A comprehensive study of CD44 rs 187115 variant and cancer risk in a central Chinese population. J Cell Biochem. 2019; 120:12949–57. 10.1002/jcb.28566 30860617

[23] Yadav A, Gupta A, Rastogi N, Agrawal S, Kumar A, Kumar V, Mittal B. Association of cancer stem cell markers genetic variants with gallbladder cancer susceptibility, prognosis, and survival. Tumour Biol. 2016; 37:1835–44. 10.1007/s13277-015-3929-6 26318430

[24] Sharma KL, Yadav A, Gupta A, Tulsayan S, Kumar V, Misra S, Kumar A, Mittal B. Association of genetic variants of cancer stem cell gene CD44 haplotypes with gallbladder cancer susceptibility in North Indian population. Tumour Biol. 2014; 35:2583–9. 10.1007/s13277-013-1340-8 24186075

[25] Verma A, Kapoor R, Mittal RD. Cluster of Differentiation 44 (CD44) Gene Variants: A Putative Cancer Stem Cell Marker in Risk Prediction of Bladder Cancer in North Indian Population. Indian J Clin Biochem. 2017; 32:74–83. 10.1007/s12291-016-0580-y 28149016 PMC5247376

[26] Stotz M, Herzog SA, Pichler M, Smolle M, Riedl J, Rossmann C, Bezan A, Stöger H, Renner W, Berghold A, Gerger A. Cancer Stem Cell Gene Variants in CD44 Predict Outcome in Stage II and Stage III Colon Cancer Patients. Anticancer Res. 2017; 37:2011–8. 10.21873/anticanres.11545 28373475

[27] Ramasami S, Kerr KM, Chapman AD, King G, Cockburn JS, Jeffrey RR. Expression of CD44v6 but not E-cadherin or beta-catenin influences prognosis in primary pulmonary adenocarcinoma. J Pathol. 2000; 192:427–32. 10.1002/1096-9896(2000)9999:9999<::AID-PATH741>3.0.CO;2-Z 11113858

[28] Qian H, Zhang D, Bao C. Two variants of Interleukin-1B gene are associated with the decreased risk, clinical features, and better overall survival of colorectal cancer: a two-center case-control study. Aging (Albany NY). 2018; 10:4084–92. 10.18632/aging.101695 30563955 PMC6326653

